# A meta-analysis of randomized controlled trials that compare standard doxorubicin with other first-line chemotherapies for advanced/metastatic soft tissue sarcomas

**DOI:** 10.1371/journal.pone.0210671

**Published:** 2019-01-10

**Authors:** Kazuhiro Tanaka, Masanori Kawano, Tatsuya Iwasaki, Ichiro Itonaga, Hiroshi Tsumura

**Affiliations:** Department of Orthopaedic Surgery, Faculty of Medicine, Oita University, Yufu City, Oita, Japan; The Chinese University of Hong Kong, HONG KONG

## Abstract

**Objective:**

The standard treatment for patients with advanced/metastatic soft tissue sarcomas (ASTS) is systemic chemotherapy with doxorubicin. A previous meta-analysis of 8 randomized controlled trials (RCTs) demonstrated the superiority of single-agent doxorubicin over doxorubicin-based combination chemotherapy for ASTS. However, meta-analyses of all RCTs that compare doxorubicin to other single-agent or combination regimens as first-line treatments for ASTS are lacking. We conducted a systematic review and meta-analysis to evaluate the efficacy and toxicity of current primary treatments for ASTS.

**Methods:**

Eligible studies were RCTs of first-line chemotherapies for ASTS comparing doxorubicin alone to other single agents or to combination therapies (experimental arm). Data from studies reporting hazard ratios (HR) and 95% confidence intervals (CI) for overall survival (OS) and progression-free survival (PFS) were pooled. Other time-to-event endpoints were extracted from the studies based on Kaplan-Meier estimates, and pooled odds ratios (OR) and 95% CI were calculated.

**Results:**

Twenty-seven eligible RCTs comprising 6156 patients were identified. Overall, the 1-year OS (OR 0.88, 95% CI 0.79–0.99, *P* = 0.03) was significantly improved in the experimental arm over the doxorubicin-only arm; however, there was no significant difference in 2-year OS (OR 0.87, 95% CI 0.73–1.03, *P* = 0.11) or OS (HR 0.97, 95% CI 0.91–1.03, *P* = 0.28) between the two groups. PFS and other time-to-event endpoints were not significantly different between the two treatment arms. While incidences of overall severe adverse events were not significantly different (OR 1.20, 95% CI 0.88–1.65, *P* = 0.26), severe nausea/vomiting was significantly more frequent in the experimental arm (OR 1.90, 95% CI 1.27–2.83, *P* = 0.002).

**Conclusion:**

The efficacies of doxorubicin-only and experimental arm regimens were similar, although toxicities were more frequent in the experimental arms. Hence, doxorubicin monotherapy remains suitable as a standard first-line regimen for ASTS.

## Introduction

Soft tissue sarcomas (STS) are rare malignant tumors that comprise approximately 1% of all malignant tumors [1]. The Soft Tissue Tumor Registry of the Japanese Orthopaedic Association had 1529 STS patients in Japan registered in 2015 [2]. The standard treatment for all localized STS is surgical resection, whereas systemic chemotherapy is the preferred treatment for patients with advanced and metastatic STS (ASTS).

The standard first-line regimen for ASTS as recommended by worldwide guidelines is doxorubicin (DOX) alone [3–5]. The efficacy of DOX against ASTS has been demonstrated by previous randomized controlled trials (RCTs), and the superiority of DOX monotherapy over combination chemotherapy was shown in a meta-analysis of 8 RCTs of first-line treatment for ASTS by Bramwell et al. in 2003 [6]. The concomitant agents used in the experimental groups of these RCTs were streptozotocin, vincristine, cyclophosphamide, dacarbazine, vindesine, ifosfamide, cisplatin, and mitomycin.

Pazopanib, the first molecular-targeted therapeutic agent for ASTS, was approved in the United States, Europe, and Japan in 2012 [7]. More recently, trabectedin, eribulin, and olaratumab were also approved for ASTS [8–10]. Therefore, several more recent RCTs comparing DOX alone with combination chemotherapy or other regimens were performed. These included RCTs comparing DOX alone to trabectedin [11–13], while another comparing DOX to pazopanib is currently ongoing [14]. Notably, the combination of olaratumab and DOX as a first-line treatment for ASTS has shown superior overall survival (OS) over DOX alone [10]. These results suggested it would be valuable to perform an updated meta-analysis of RCTs for ASTS, including the modern trials of new agents.

In this meta-analysis of 27 RCTs, we compared the efficacy of DOX monotherapy with that of other single-agent and combination chemotherapy regimens for the first-line treatment of ASTS.

## Methods

### Study selection

PubMed, Scopus, EBSCOhost MEDLINE, and the Cochrane Central Register of Controlled Trials were searched in accordance with the Preferred Reporting Items for Systematic Reviews and Meta-Analyses (PRISMA) guidelines [15]. The search algorithm followed the method previously described [16], except for the inclusion of the keywords ‘doxorubicin or adriamycin or anthracycline’ and ‘first line or first-line’. We included phase II and III RCTs of first-line systemic therapies for ASTS that compared single-agent DOX with other chemotherapy regimens and were published in English between January 1974 and September 2018. RCTs investigating bone sarcoma, rhabdomyosarcoma, other pediatric sarcomas, Kaposi sarcoma, and gastrointestinal stromal tumors were excluded owing to the distinct biological characteristics and treatment strategies for these tumors. Reviews, meta-analyses, and non-randomized clinical trials were also excluded. All studies retrieved by the search were independently screened and crosschecked according to the above eligibility criteria by 2 authors (KT and MK). In case of discrepancy, a third author (TI or II) was consulted.

### Data extraction

Data extracted from eligible RCTs included publication date; study phase; primary and secondary endpoints; dose of standard-arm DOX; regimen and dose of the experimental arm; presence of intention-to-treat (ITT) analysis; sample size; and patient age, sex, and performance status. The following were also recorded: sarcoma subtypes, histologic grades, number of patients with advanced or metastatic disease, number of patients with prior radiotherapy, response rates (RRs), PFS (or time-to-progression [TTP]), OS, severe (grade 3 or higher) adverse events (AEs), and descriptions of post-protocol treatment. For survival data, medians, hazard ratios (HRs), confidence intervals (CIs), and *P*-values were extracted. The RR was defined as the proportion of patients assessed as having achieved complete or partial response based on the criteria described in each study. Three-month (or 12-week) PFS, 6-month (or 24-week) PFS, 1-year PFS, 1-year OS, and 2-year OS based on Kaplan-Meier (KM) estimates were extracted from the studies. When these data were not described in the articles, PFS or OS KM curves were used to calculate estimations as binary proportions.

### Statistical analysis

In the meta-analyses, pooled odds ratios (ORs) and corresponding 95% CIs were calculated for RR; 3-month, 6-month, and 1-year PFS; 1- and 2-year OS; and AEs. Additionally, pooled HRs and 95% CIs were calculated for PFS and OS using the Mantel-Haenszel and inverse variance random or fixed effects model. A random effects model was applied if the *P*-value for the heterogeneity test was less than 0.1. Heterogeneity among study results was quantified using Cochrane’s Q-test and I^2^ statistics. Primary and major secondary endpoints of the present study were OS and PFS, respectively, based on the previous surrogacy analysis of endpoint [16]. The risk of bias in the included studies was assessed using the Cochrane Risk of Bias Assessment Tool, and publication bias was evaluated using a funnel plot. Meta-analyses were performed using Review Manager (RevMan), version 5.3 (Nordic Cochrane Centre, Cochrane Collaboration, Copenhagen, Denmark). Other statistical analyses were performed using SAS, version 9.4 (SAS Institute, Cary, NC, USA). All statistical tests were 2-sided, and *P-*values ≤0.05 were considered statistically significant.

## Results

### Characteristics of eligible studies

**Fig 1:**
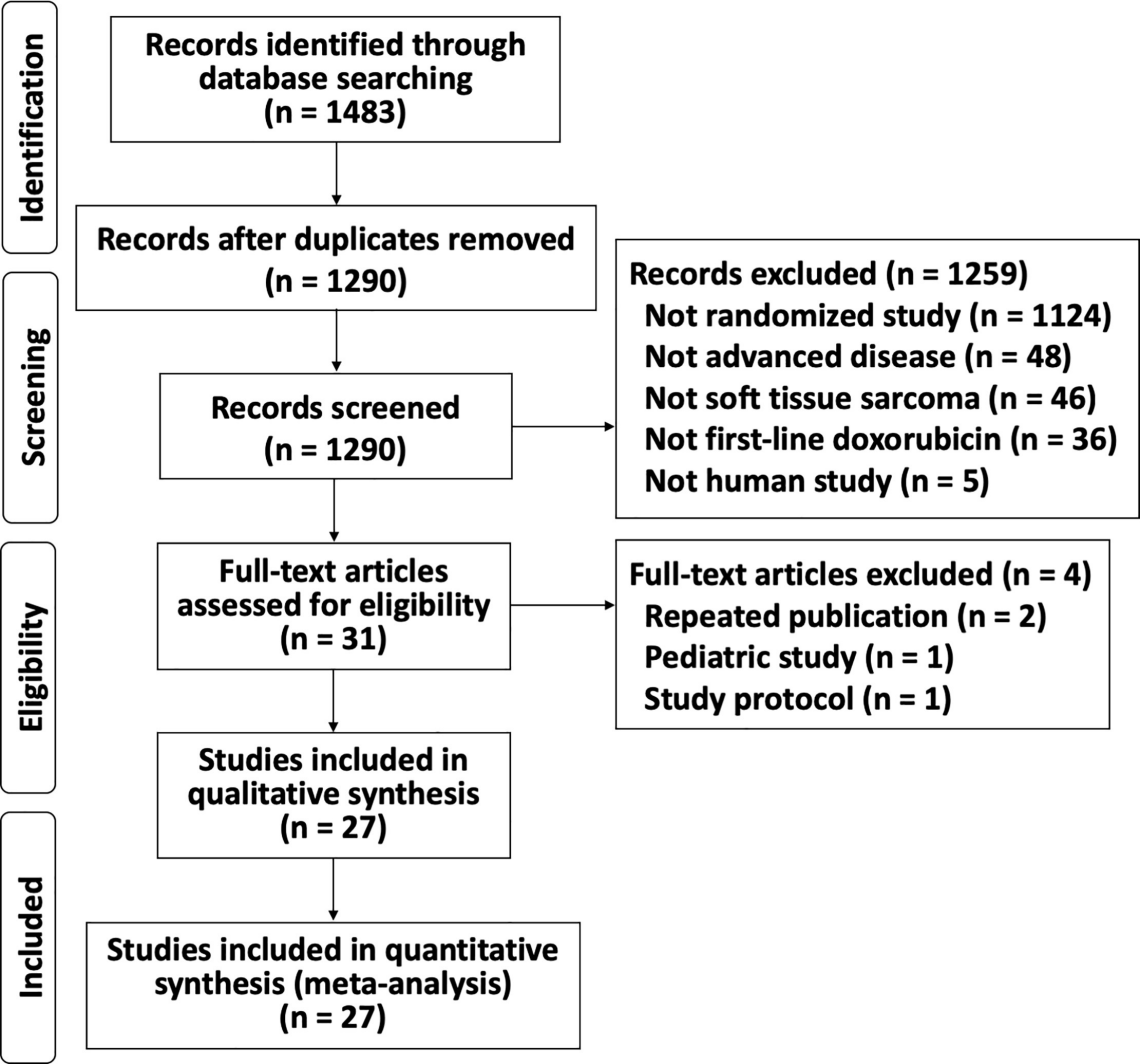
PRISMA flow diagram. PRISMA, Preferred Reporting Items for Systematic Reviews and Meta-Analyses.

**Fig 2:**
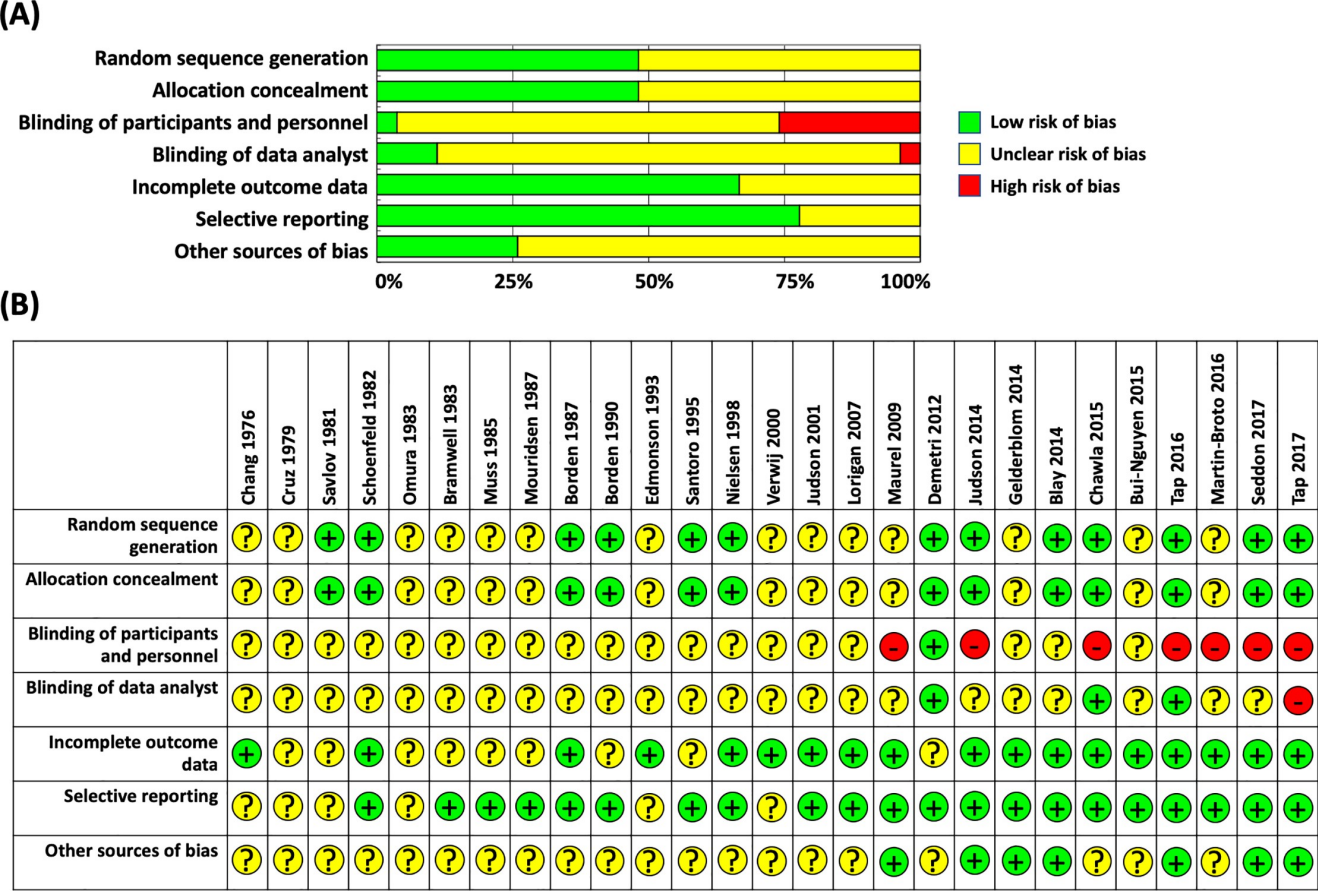
Assessment of the risk of bias of the included studies. (A) Risk of bias graph. (B) Risk of bias summary.

**Fig 3:**
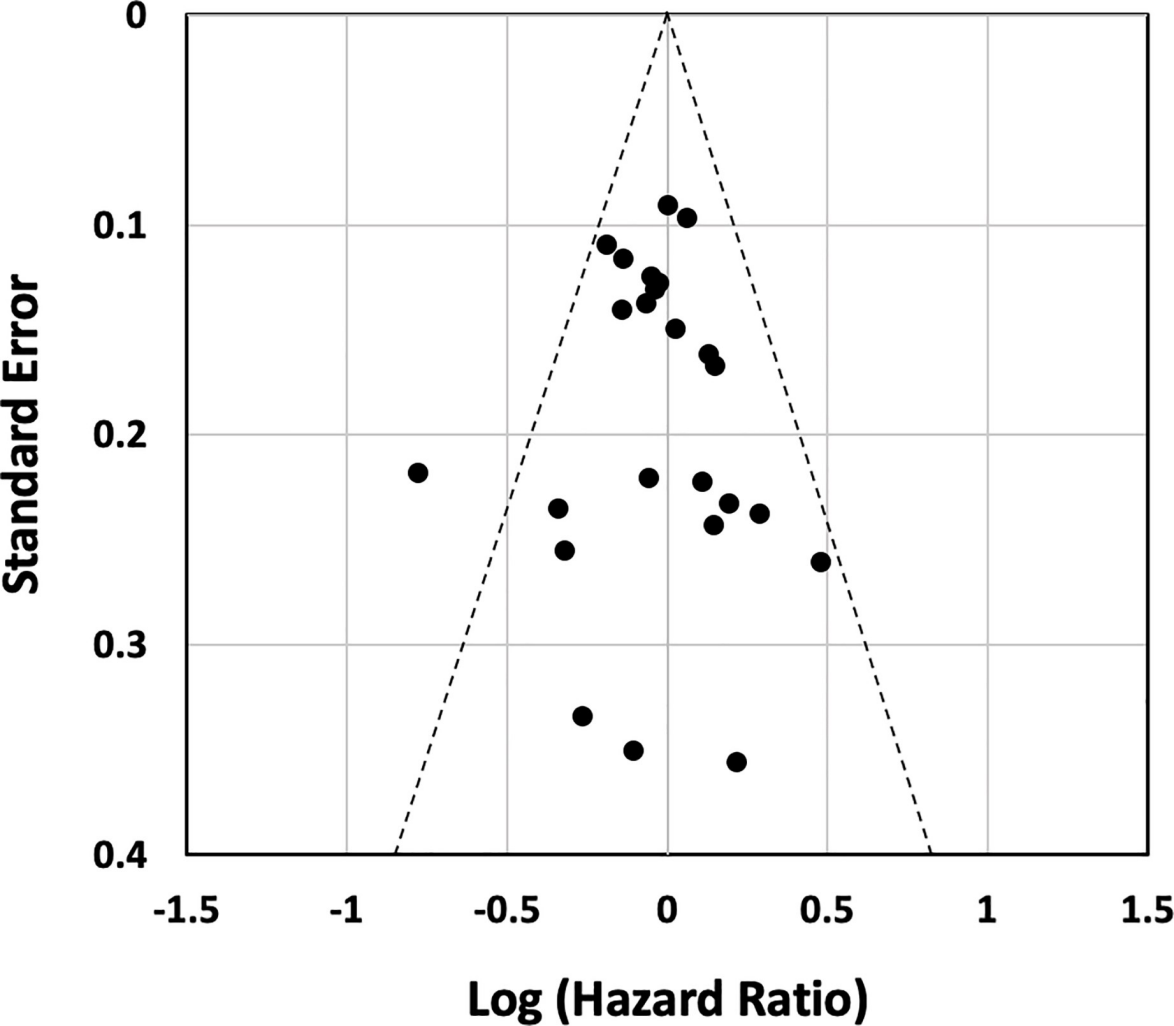
Funnel plot of the including studies evaluating the presence of publication bias.

The search initially unearthed 1483 articles. After eliminating duplicates, 1290 abstracts were further screened and 1259 studies were excluded because they were not RCTs, described cancers other than sarcomas or STS, did not describe advanced/metastatic diseases, were non-human studies, or did not use DOX alone as the first-line standard treatment. The full texts of the remaining 31 articles were further evaluated, and 2 duplicate publications, 1 study protocol-only paper, and 1 pediatric population study were also excluded. Ultimately, 27 RCTs were included in the final analysis (Fig 1) [10–13,17–39]. The characteristics of the 27 eligible RCTs are summarized in Tables 1 and 2. Due to the difficulty of masking of the treatment by intra-venous infusion of chemotherapeutic drugs, risk of bias for blinding of participants and personnel and outcome assessment were found across studies. Moreover, many studies did not described detail about random sequence generation and allocation concealment (Fig 2). Although there was some asymmetry of a small study with outlier, there was no strong evidence of publication bias for RCTs of first-line DOX for ASTS based on the funnel plot (Fig 3).

**Table 1:** Characteristics of RCTs.

	RCTs, overall			Treatment in experimental arm			
				Combination chemotherapy with DOX		Other regimens without DOX	
	No. of studies (%)	No. of patients (%)	Median no. of patients	No. of studies (%)	No. of patients (%)	No. of studies (%)	No. of patients (%)
	27 (100)	6156 (100)	133	14 (100)	3954 (100)	13 (100)	2202 (100)
Trial phase							
II	10 (37.0)	1137 (18.5)	122	4 (28.6)	508 (12.8)	6 (46.2)	629 (28.6)
III	11 (40.7)	3534 (57.4)	279	5 (35.8)	2169 (54.9)	6 (46.2)	1365 (62.0)
Not specified	6 (22.2)	1485 (24.1)	268	5 (35.8)	1277 (32.3)	1 (7.6)	208 (9.4)
Primary endpoint							
OS	2 (7.4)	1095 (17.8)	NA	2 (14.3)	1095(27.7	0	0
Other time-to-event (PFS, 3m-PFS, etc)	10 (37.0)	1589 (25.8)	130	4 (28.6)	508 (12.8)	6 (46.2)	1081 (49.1)
RR	1 (3.7)	95 (1.5)	N	0	0	1 (7.6)	95 (4.3)
Not specified	14 (51.9)	3377 (54.9)	215.5	8 (57.1)	2351 (59.5)	6 (46.2)	1026 (46.6)
ITT analysis included							
Yes	9 (33.3)	2075 (33.7)	133	4 (28.6)	1343 (34.0)	5 (38.5)	732 (33.2)
No	18 (66.7)	4081 (66.3)	209	10 (71.4)	2611 (66.0)	8 (61.5)	1470 (66.8)
Post-protocol treatment described							
Yes	14 (51.9)	2897 (47.1)	132.5	7 (50.0)	1824 (46.1)	8 (61.5)	1188 (54.0)
No	13 (48.1)	3259 (52.9)	279	7 (50.0)	2130 (53.9)	5 (38.5)	1014 (46.0)

A phase II/III study was counted as phase III study. Abbreviations: RCT, randomized controlled trial; DOX, doxorubicin; OS, overall survival; PFS, progression-free survival; 3m-PFS, 3 month-PFS; RR, response rate; ITT, intention-to-treat.

**Table 2:** Description of RCTs.

Study	No. of patients	Experimental regimen	Study phase	Primary Endpoint	ITT analysis	Post-protocol treatment
**RCTs comparing DOX and DOX-based combination chemotherapy**						
Chang 1976 [17]	33	DOX+Streptozotocin	not specified	not specified	not specified	not specified
Schoenfeld 1982 [18]	221	1) VCR+DOX+CPA 2) VCR+Act-D+CPA	not specified	not specified	not specified	Crossover
Omura 1983 [19]	315	DOX+DTIC	not specified	not specified	not specified	not specified
Muss 1985 [20]	132	DOX+CPA	III	not specified	not specified	not specified
Borden 1987 [21]	361	DOX+DTIC	not specified	not specified	not specified	not specified
Borden 1990 [22]	347	DOX+Vindesine	not specified	not specified	not specified	not specified
Edmonson 1993 [23]	279	1) DOX+IFM 2) DOX+MMC+CDDP	III	not specified	not specified	not specified
Santoro 1995 [24]	663	1) DOX+IFM 2) CYVADIC	III	not specified	not specified	not specified
Maurel 2009 [25]	132	DOX+IFM	II	PFS	not specified	IFM, DTIC, GEM+DTIC
Demetri 2012 [26]	128	DOX+Conatumumab	II	PFS	not specified	Roll over
Judson 2014 [27]	455	DOX+IFM	III	OS	+	DOX, EPI, IFM, TRAB, PAZ, ERIB, DTIC, GEM+DOC, etc
Tap 2016 [10]	133	DOX+Olaratumab	II	PFS	+	DOX, GEM+DOC, TRAB, PAZ, ERIB, GEM, DTIC, DOC, etc
Martin-Broto 2016 [13]^)^	115	DOX+TRAB	II	PFS	+	not specified
Tap 2017 [28]	640	DOX+Evofosfamide	III	OS	+	DOX, IFM, TRAB, GEM+DOC, PAZ, ERIB, GEM, DTIC, etc
**RCTs comparing DOX and other chemotherapy without DOX**						
Cruz1979 [29]	117	1) Act-D+LPAM 2) Act-D+LPAM+VCR 3) Act-D+LPAM+NSC1026	III	not specified	not specified	Crossover
Savlov 1981 [30]	208	Cycloleucine	not specified	not specified	not specified	Crossover
Bramwell 1983 [31]	71	Carminomycin	II	not specified	not specified	Crossover
Mouridsen 1987 [32]	210	EPI	II/III	not specified	not specified	Crossover
Nielsen 1998 [33]	334	EPI	III	not specified	not specified	not specified
Verweij 2000 [34]	86	DOC	II	not specified	not specified	Crossover
Judson 2001 [35]	95	Liposomal doxorubicin	II	RR	+	not specified
Lorigan 2007 [36]	326	IFM	III	PFS	not specified	not specified
Gelderblom 2014 [37]	118	Brostallicin	II	26-week PFR	not specified	DOX-based, IFM, etc
Blay 2014 [11]	121	TRAB	III	PFS	+	TRAB, etc
Bui-Nguyen 2015 [12]	133	TRAB	II	PFS	+	not specified
Chawla 2015 [38]	123	Aldoxorubicin	II	PFS	+	not specified
Seddon 2017 [39]	257	GEM+DOC	III	24-week PFR	+	DOX, IFM, TRAB, PAZ, GEM+DOC, GEM, etc

Abbreviations: RCT, randomized controlled trial; OS, overall survival; PFS, progression-free survival; PFR, progression-free rate; RR, response rate; ITT, intention-to-treat; DOX, doxorubicin; VCR, vincristine; CPA, cyclophosphamide; Act-D, actinomycin D; DTIC, dacarbazine; IFM, ifosfamide; MMC, mitomycin C; CDDP, cisplatin; CYVADIC, CPA+VCR+DOX+DTIC; TRAB, trabectedin; LPAM, melphalan; EPI, epirubicin; DOC, docetaxel; GEM, gemcitabine; PAZ, pazopanib; ERIB, eribulin.

Altogether, 6156 patients were randomly assigned to experimental or DOX-only arms, which included 3371 and 2785 patients, respectively. The median number of patients per RCT was 133. All 27 RCTs had single-agent DOX as the control arm. After excluding 1 older study with a DOX dose of 1.2 mg/kg [29], the median DOX dose in the control arms of the remaining studies was 75 mg/m^2^ (range 60–80 mg/m^2^). Among 32 experimental arms in 27 RCTs, 30 consisted of cytotoxic drugs (either single-agent or combination) and 2 included molecular-targeted drugs. Ten RCTs were phase II and 11 were phase III. Six RCTs did not specify their study phases. Primary endpoint and ITT analyses were defined in 13 (48.1%) and 9 (33.3%) RCTs, while post protocol treatments were described in 14 (51.9%). For OS, HR was described in 11RCTs (40.7%) and estimated using KM curve in 13 RCTs (48.1%), while there was no OS data in the remaining 3 RCTs. On the other hand, HR for PFS was described in 11 RCTs (40.7%) and estimated using KM curve in 12 RCTs (44.4%). PFS data was not shown in the remaining 4 RCTs.

In the 14 RCTs investigating combination chemotherapy with DOX, a total of 3954 patients were randomly assigned (Table 1); there were 4 and 5 phase II and III studies, respectively. Primary endpoint and ITT analyses were described in 6 and 4 of the 14 RCTs, respectively.

### Meta-analysis of efficacy

**Fig 4:**
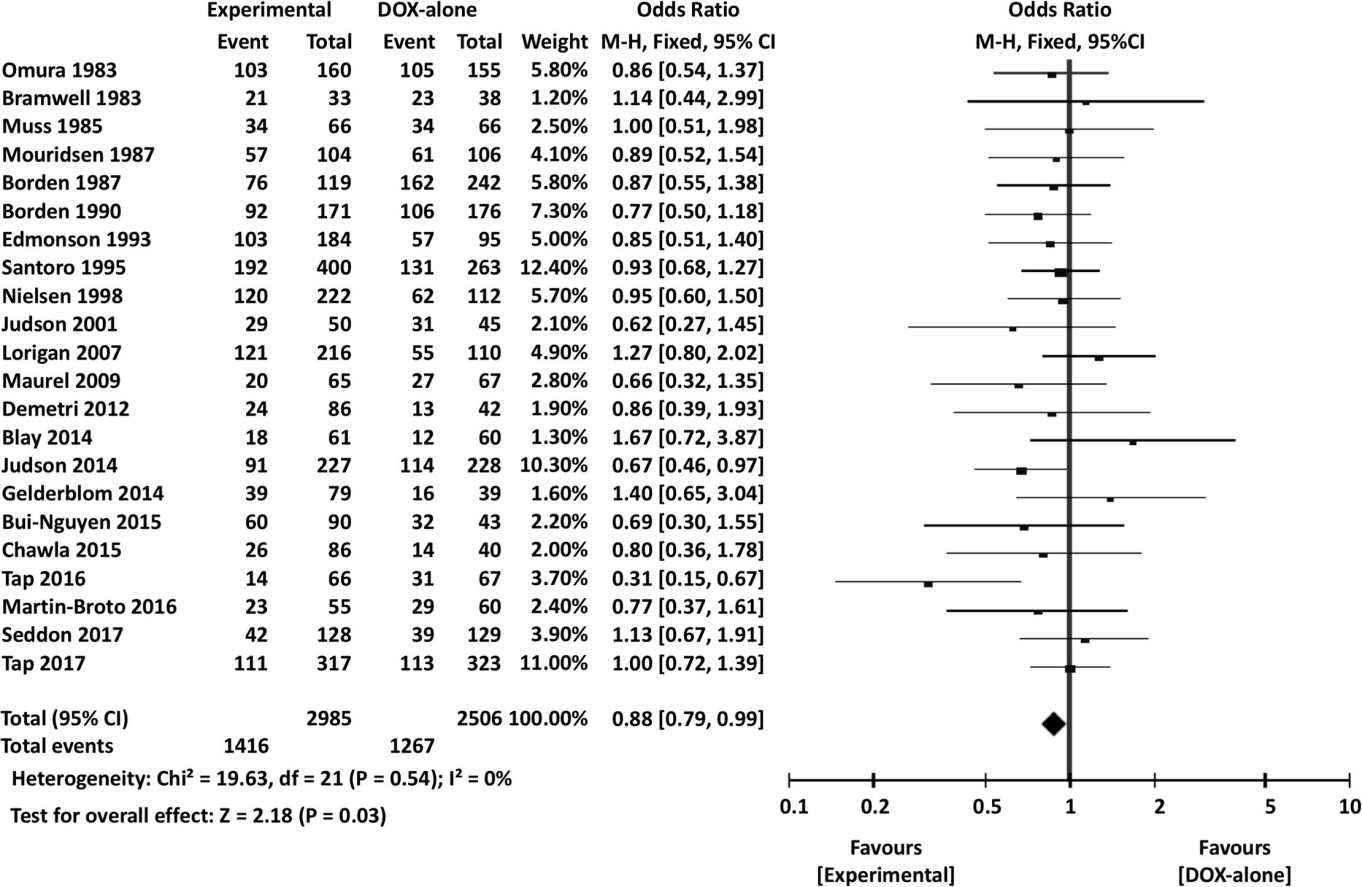
Comparisons of doxorubicin alone vs experimental chemotherapy: Forest plots of 1-year overall survival. M-H, Mantel-Haenszel; CI, confidence interval.

**Fig 5:**
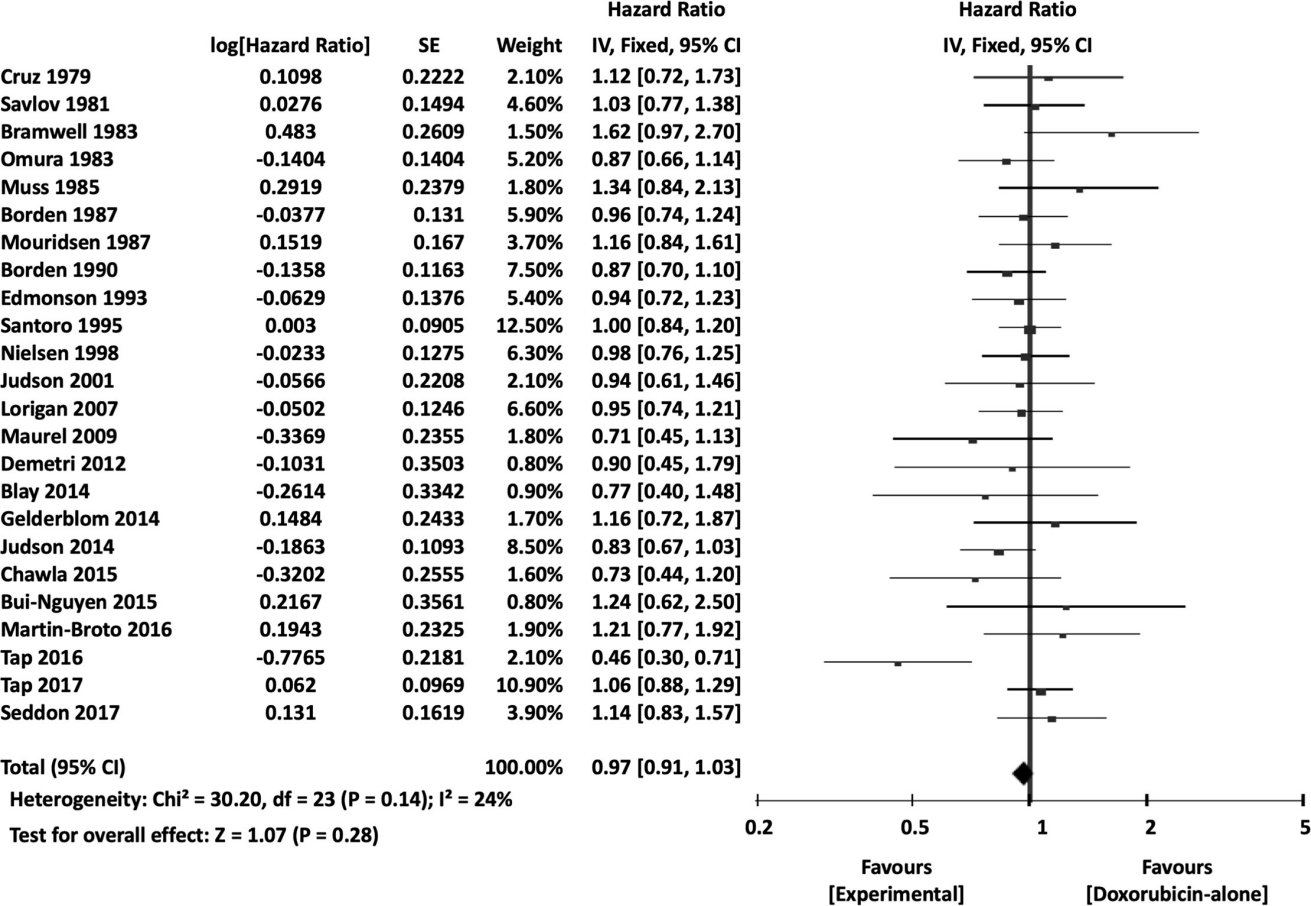
Comparisons of doxorubicin alone vs experimental chemotherapy: Forest plots of overall survival. M-H, Mantel-Haenszel; CI, confidence interval.

The meta-analysis results are summarized in Table 3. Overall, the experimental arm demonstrated significantly better 1-year OS (OR 0.88, 95% CI 0.79–0.99, *P* = 0.03) (Fig 4). However, there were no significant differences between DOX single-agent and experimental arms in terms of 2-year OS (OR 0.87, 95% CI 0.73–1.03, *P* = 0.11) or overall OS (HR 0.97, 95% CI 0.91–1.03, *P* = 0.28) (Fig 5).

**Table 3:** Summary of the meta-analysis.

Endpoint	All RCTs		RCTs comparing DOX vs DOX-based combination therapy	
	HR/OR (95% CI)	*P*	HR/OR (95% CI)	*P*
OS	0.97 (0.91–1.03)	0.28	0.92 (0.82–1.03)	0.13
1-year OS	0.88 (0.79–0.99)	0.03	0.82 (0.72–0.94)	0.004
2-year OS	0.87 (0.73–1.03)	0.11	0.84 (0.67–1.05)	0.14
PFS	1.02 (0.91–1.13)	0.74	0.91 (0.85–0.99)	0.02
3-month PFS	1.11 (0.85–1.46)	0.43	0.77(0.58–1.01)	0.06
6-month PFS	0.91 (0.73–1.15)	0.44	0.81 (0.61–1.06)	0.13
1-year PFS	0.88 (0.69–1.13)	0.33	0.77 (0.64–0.91)	0.003
2-year PFS	0.88 (0.70–1.09)	0.23	1.04 (0.81–1.33)	0.78
RR	1.11 (0.85–1.46)	0.45	0.76 (0.60–0.97)	0.03
AEs, overall	1.20 (0.88–1.65)	0.26	1.81 (1.35–2.43)	<0.0001
Nausea/vomiting	1.90 (1.27–2.83)	0.002	2.52 (1.47–4.33)	0.0008
Leukopenia	1.17 (0.72–1.89)	0.52	2.51 (2.00–3.16)	<0.00001
Neutropenia	0.79 (0.52–1.21)	0.28	1.08 (0.61–1.93)	0.79

Abbreviations: RCT, randomized controlled trial; DOX, doxorubicin; HR, hazard ratio; OR, odds ratio; CI, confidence interval; OS, overall survival; PFS, progression-free survival; RR, response rate; AEs, adverse events.

**Fig 6:**
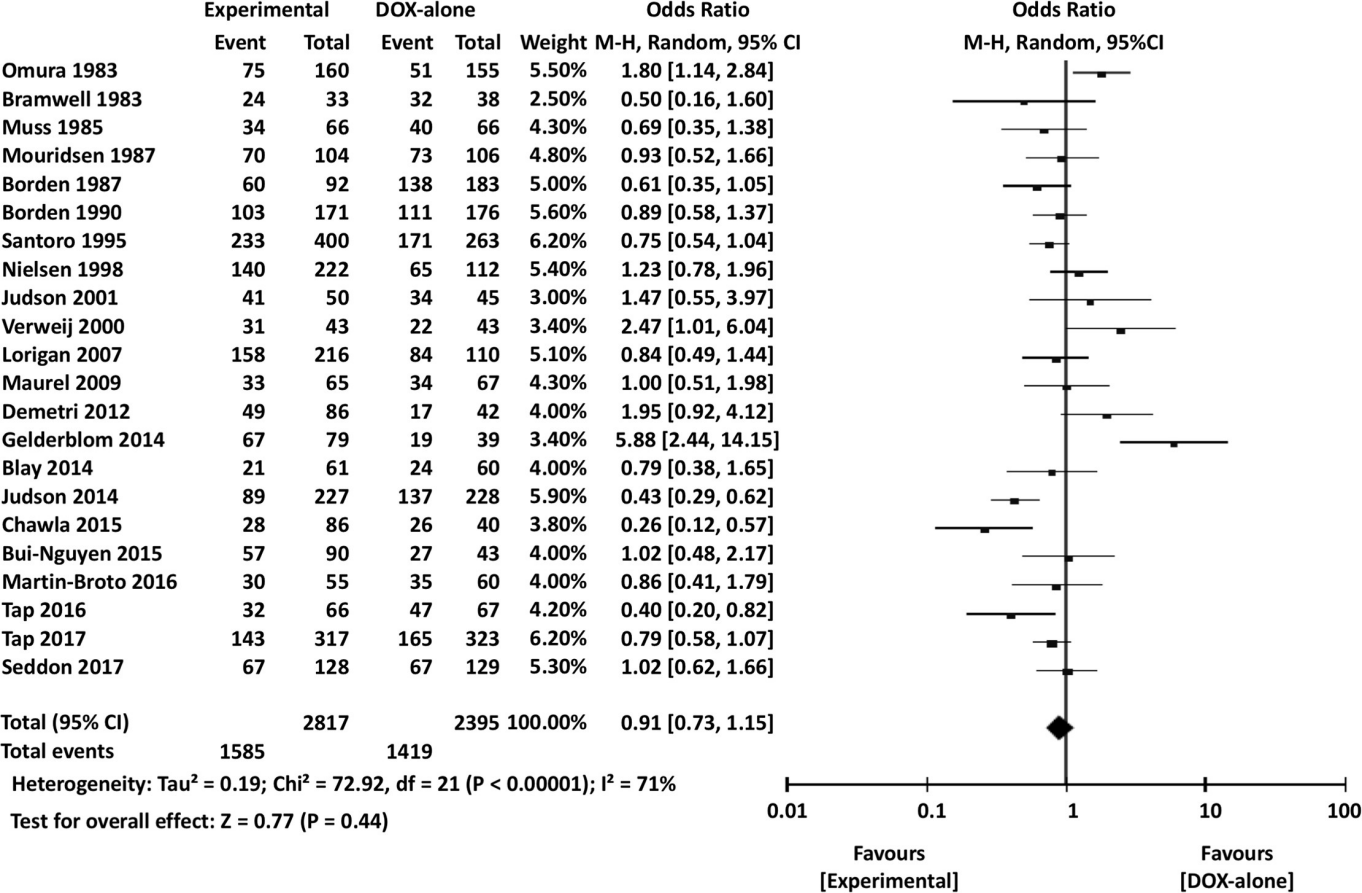
Comparisons of doxorubicin alone vs experimental chemotherapy: Forest plots of 6-month progression-free survival. CI, confidence interval; M-H, Mantel-Haenszel.

**Fig 7:**
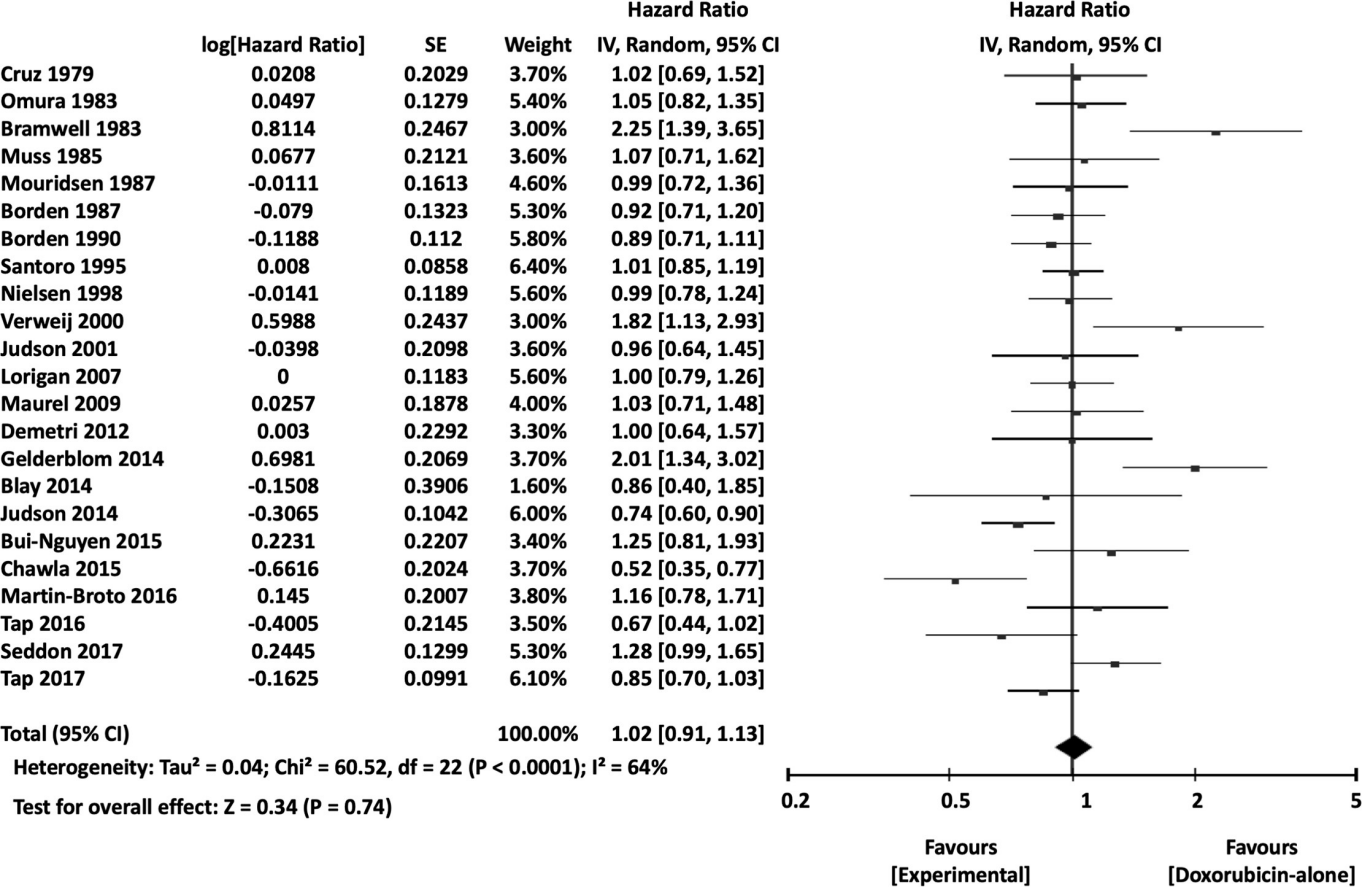
Comparisons of doxorubicin alone vs experimental chemotherapy: Forest plots of progression-free survival. CI, confidence interval; M-H, Mantel-Haenszel.

Our analyses revealed no significant differences between control and experimental arms in terms of 3-month PFS (OR 1.11, 95% CI 0.85–1.46, *P* = 0.43), 6-month PFS (OR 0.91, 95% CI 0.73–1.15, *P* = 0.44) (Fig 6), 1-year PFS (OR 0.88, 95% CI 0.69–1.13, *P* = 0.33), 2-year PFS (OR 0.88, 95% CI 0.70–1.09, *P* = 0.23), overall PFS (HR 1.02, 95% CI 0.91–1.13, *P* = 0.74) (Fig 7), or RR (OR 1.11, 95% CI 0.85–1.46, *P* = 0.45).

### Meta-analysis of adverse events

**Fig 8:**
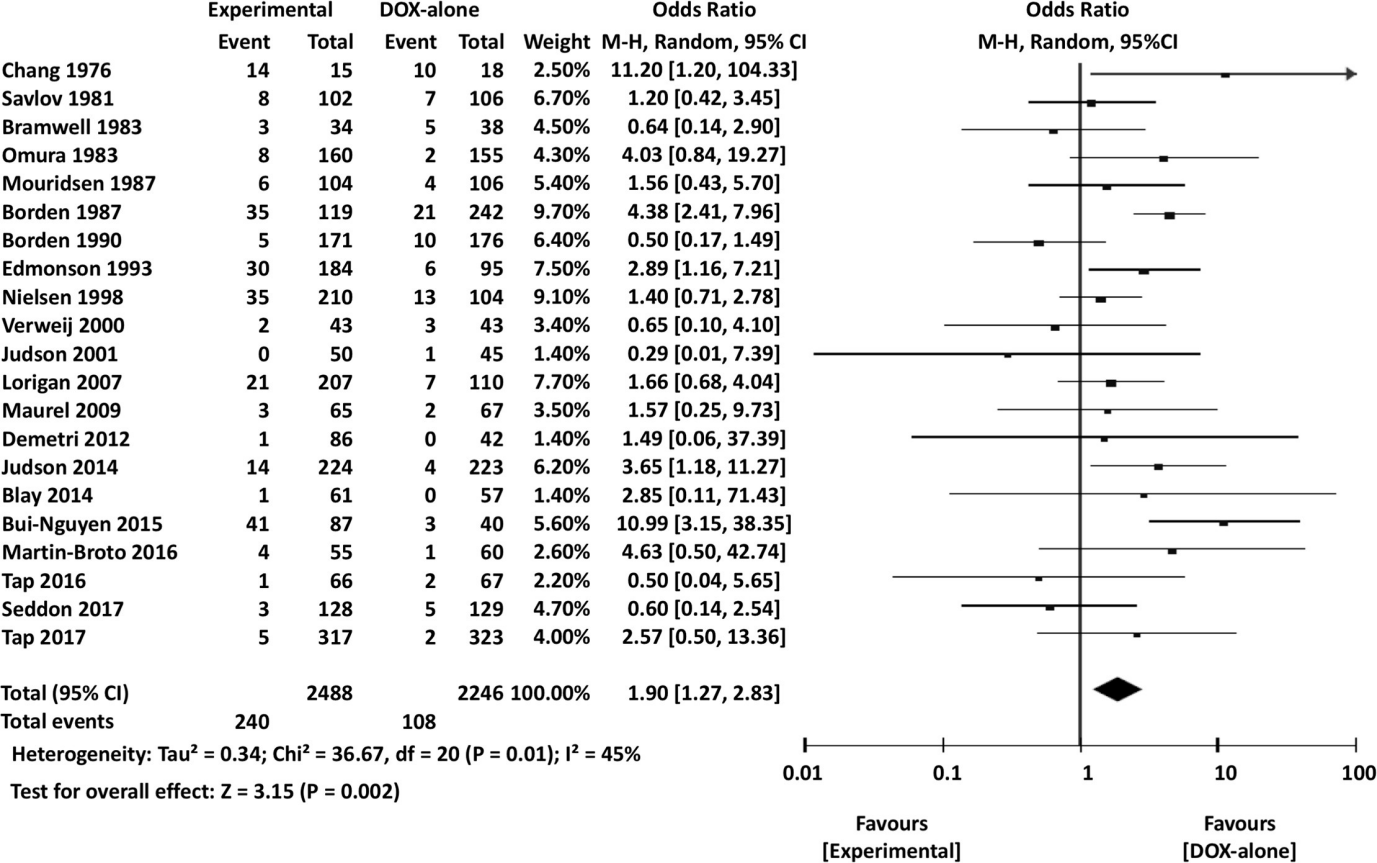
Comparisons of doxorubicin alone vs experimental chemotherapy: Forest plots of the incidence of severe nausea/vomiting adverse events. CI, confidence interval; M-H, Mantel-Haenszel.

The incidences of overall severe AEs (grades 3 or higher) were not significantly different between experimental and DOX-only arms (OR 1.20, 95% CI 0.88–1.65, *P* = 0.26). There was also no significant difference in the occurrence of severe leukopenia (OR 1.17, 95% CI 0.72–1.89, *P* = 0.52) or neutropenia (OR 0.79, 95% CI 0.52–1.21, *P* = 0.28) between DOX-only and experimental arms. However, severe nausea or vomiting was significantly less frequent in DOX-only arms than in experimental arms (OR 1.90, 95% CI 1.27–2.83, *P* = 0.002) (Fig 8).

### Meta-analysis of RCTs comparing DOX alone and DOX-based combination chemotherapy

**Fig 9:**
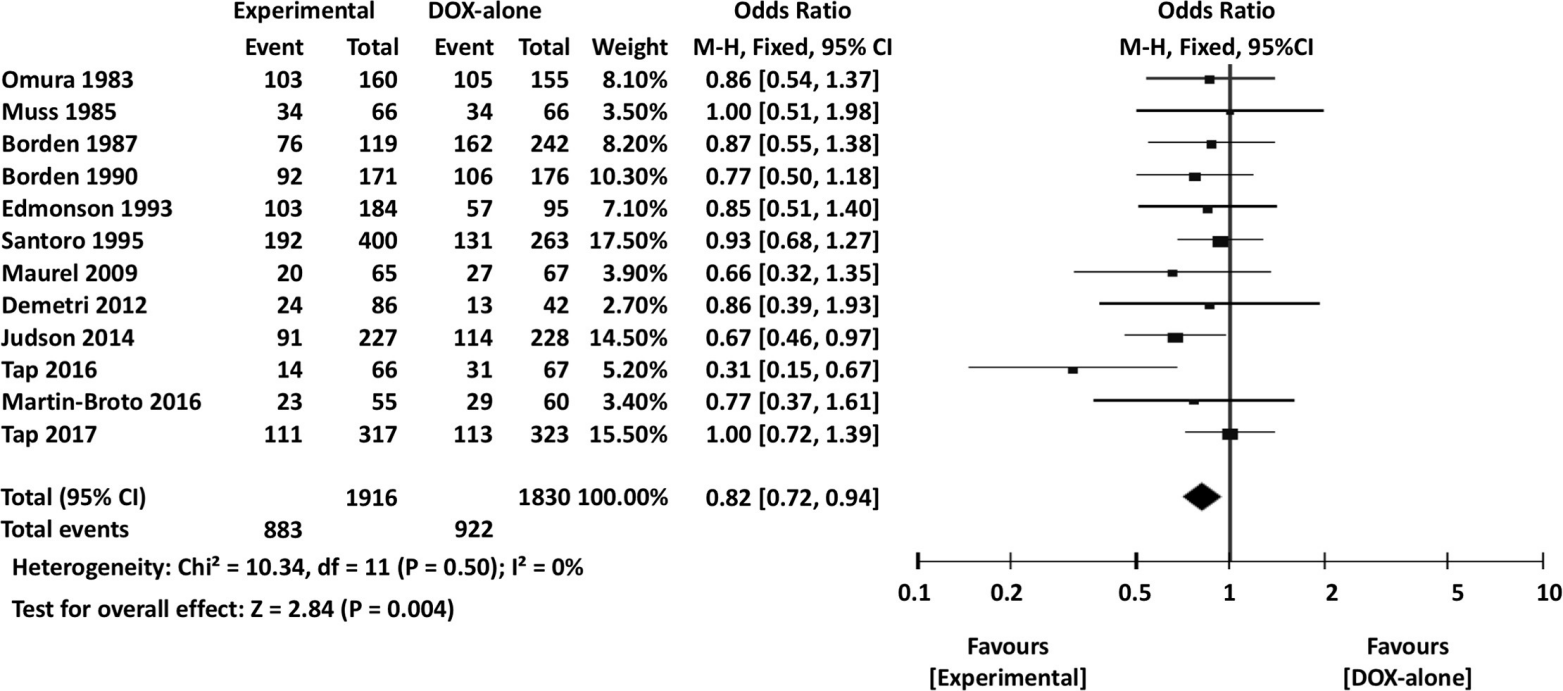
Comparisons of doxorubicin alone vs doxorubicin-based combination chemotherapy: Forest plots of 1-overall survival. M-H, Mantel-Haenszel; CI, confidence interval.

Next, subgroup meta-analyses of 14 RCTs comparing DOX alone to DOX-based combination regimens were performed. As in the overall analysis, the 1-year OS was significantly longer in combination chemotherapy arms than in DOX-only arms (OR 0.82, 95% CI 0.77–0.94, *P* = 0.004) (Fig 9). On the other hand, DOX and experimental arms did not have significantly different 2-year OS (OR 0.84, 95% CI 0.67–1.05, *P* = 0.14) or overall OS (HR 0.92, 95% CI 0.82–1.03, *P* = 0.13).

**Fig 10:**
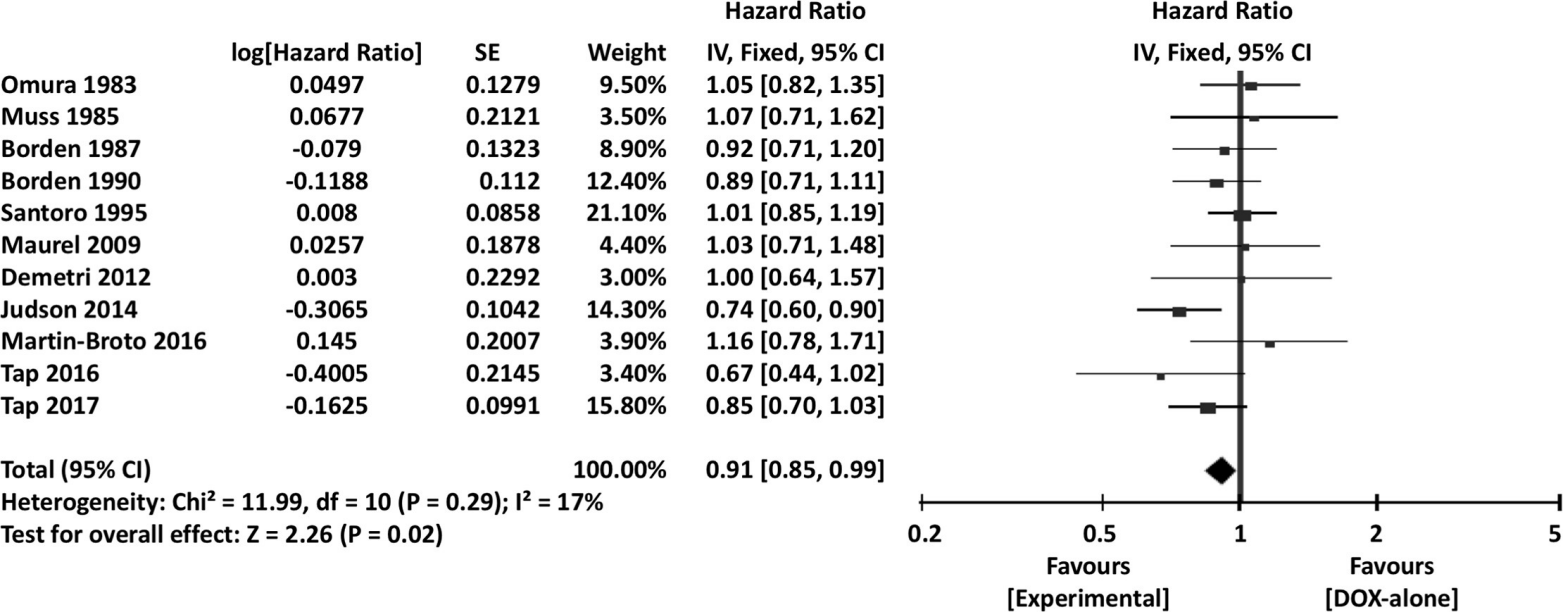
Comparisons of doxorubicin alone vs doxorubicin-based combination chemotherapy: Forest plots of progression-free survival. SE, standard error; IV, inverse variance; CI, confidence interval.

When the surrogate endpoints were analyzed, 1-year PFS (OR 0.77, 95% CI 0.64–0.91, *P* = 0.003), overall PFS (HR 0.91, 95% CI 0.85–0.99, *P* = 0.02) (Fig 10), and RR (OR 0.76, 95% CI 0.60–0.97, *P* = 0.03) were significantly more favorable in the combination chemotherapy groups. Additional meta-analyses of the 3-month PFS (OR 0.77, 95% CI 0.58–1.01, *P* = 0.06), 6-month PFS (OR 0.81, 95% CI 0.61–1.06, *P* = 0.13), and 2-year PFS (OR 1.04, 95% CI 0.81–1.33, *P* = 0.78) showed no significant differences between DOX-only and combination therapy arms.

**Fig 11:**
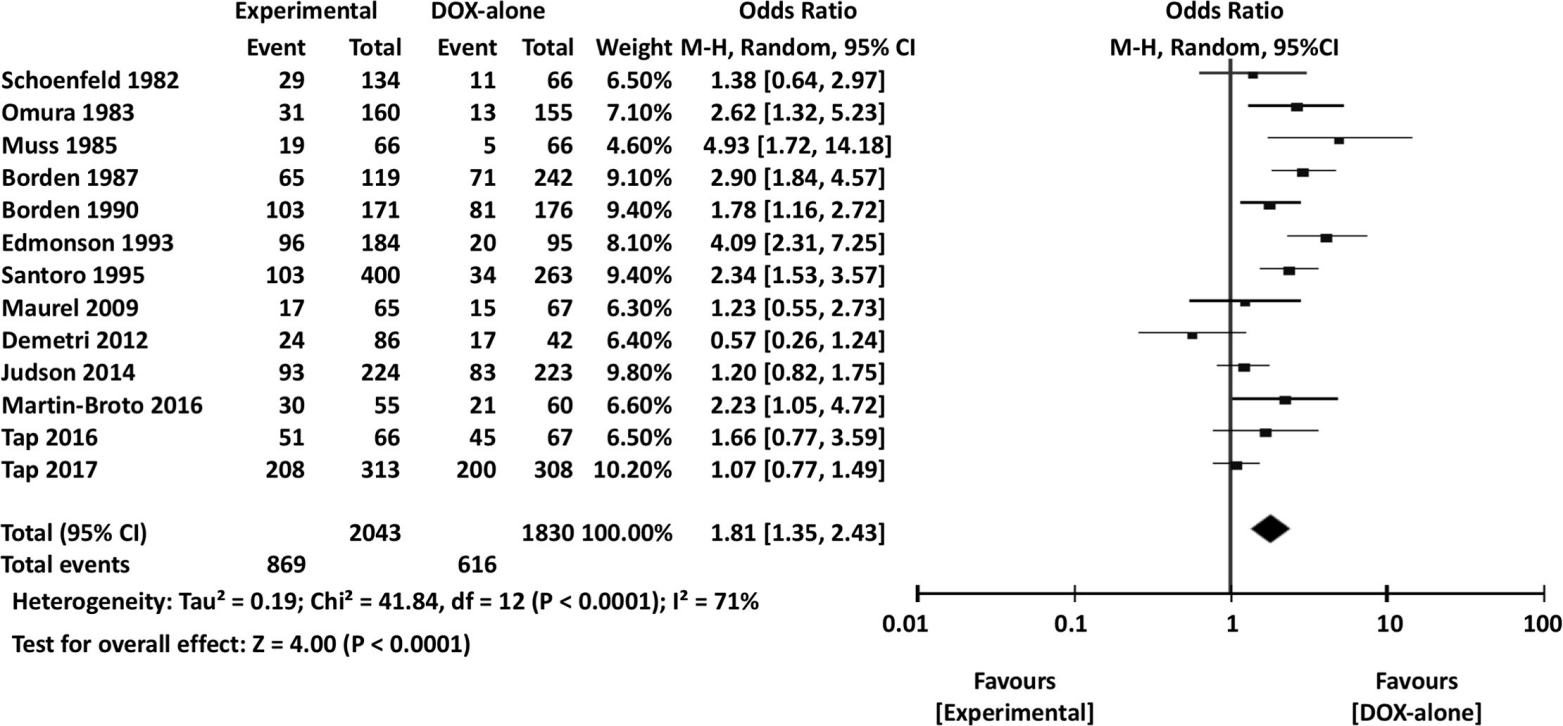
Comparisons of doxorubicin alone vs doxorubicin-based combination chemotherapy: Forest plots of the overall incidence of severe adverse events. M-H, Mantel-Haenszel; CI, confidence interval.

Overall severe AEs (OR 1.81, 95% CI 1.35–2.43, *P*<0.0001) (Fig 11), leukopenia (OR 2.51, 95% CI 2.00–3.16, *P*<0.00001), and nausea or vomiting (OR 2.52, 95% CI 1.47–4.33, *P* = 0.0008) were significantly less frequent in DOX-only arms than in combination therapy arms. There were no significant differences in the incidences of severe neutropenia between DOX-only and experimental arms (OR 1.08, 95% CI 0.61–1.93, *P* = 0.79).

## Discussion

Bramwell et al.’s meta-analysis collected 8 RCTs that compared DOX alone to DOX-based combination chemotherapy for treating ASTS [6]. Subsequently, 6 similar RCTs have been conducted, as well as 13 additional RCTs of primary therapy for ASTS that compared DOX alone to other single agents or combination regimens without DOX. Ours is the first meta-analysis of the abovementioned 27 RCTs of first-line chemotherapy with standard DOX for ASTS.

Bramwell et al.’s meta-analysis included 10 DOX-based combination chemotherapy regimens administered in 8 studies, as well as 9 single-agent DOX standard arms in 8 RCTs. Two of the 8 RCTs demonstrated significantly better RRs in the combination arm than in the DOX-only arm. None of the RCTs exhibited significant differences in 1-year and 2-year mortality rates between the 2 treatment groups. Bramwell et al.’s meta-analysis revealed no significant differences in RR (OR 1.26, 95% CI 0.96–1.67, *P* = 0.10), death at 1 year (OR 0.87, 95% CI 0.73–1.05, *P* = 0.14), and death at 2 years (OR 0.84, 95% CI 0.67–1.05, *P* = 0.13) between DOX-only and DOX-based combination regimens [6]. However, other time-to-event endpoints such as overall and 3-month PFS were not analyzed in their study. On the other hand, AEs including nausea/vomiting and hematologic toxicities tended to be frequent for combination regimens, although the differences in overall AE rates among the 8 RCTs were not statistically analyzed. Therefore, their meta-analysis concluded that single-agent DOX was a suitable standard treatment for chemotherapy-naive patients with ASTS; this has remained the case in worldwide guidelines [3–5].

Conatumumab, ifosfamide, trabectedin, evofosfamide, and olaratumab were used in combination with DOX in 6 similar RCTs performed after Bramwell et al.’s meta-analysis. The combination of DOX and olaratumab significantly prolonged OS over DOX alone. In a randomized phase II study, OS was significantly better with the combination therapy (HR 0.46, 95% CI 0.30–0.71, *P* = 0.0003), although the number of patients was small (67 in the DOX arm and 66 in the DOX plus olaratumab arm) [10].

The present meta-analysis demonstrated that RR and PFS were significantly improved with the combination therapy compared to DOX alone, suggesting that RR and PFS have improved since the RCTs investigated in Bramwell et al.’s study. However, there was no significant difference in OS between the 2 groups. On the other hand, severe overall AEs, leukopenia, and nausea/vomiting rates were significantly higher in patients receiving the combination regimens. Therefore, in agreement with Bramwell et al.’s conclusion, our meta-analysis of 14 RCTs comparing DOX to combination therapy revealed that DOX alone ought to remain the recommended first-line regimen for patients with ASTS.

Recently, a meta-analysis of 22 RCTs of single agents and combination therapies for ASTS found that OS (HR 0.79, 95% CI 0.65–0.97, *P* = 0.02) and PFS (OR 0.86, 95% CI 0.73–1.00, *P* = 0.05) were significantly improved in patients receiving the combination regimens [40]. However, the actual numbers of the RCTs analyzed for OS and PFS were only 7 and 11, respectively. No RCT published before 2008 was involved in the analysis. Their study further included study abstracts, although the results were often different from those in the fully published articles, and also included studies using cytostatic/biological agents only. Moreover, the lines of treatment and patient backgrounds in each RCT were different, while the control regimens in each also varied. These caveats suggest that the results of their study ought to be interpreted with greater caution.

The limitations of our study are as follows: 1) The present meta-analysis was based only on published data, as we were unable to access individual data of the patients included in each RCT; 2) several older studies included certain subjects, such as those with mesothelioma and bone tumors, who were excluded from more recent trials; 3) several studies did not define their time-to-event endpoints; 4) the patient characteristics among the RCTs, such as histologic grade, subtypes, and proportions of metastatic and unresectable tumors, varied; and 5) some studies included a small number of patients who had received prior chemotherapy (175 out of 6156 patients: 2.8%). These limitations should be noted for interpretation of the results of the study.

Currently, a phase III trial of DOX plus olaratumab is being conducted; if the results of this trial will be in agreement with the randomized phase II trial by Tap et al. [10], there is a possibility that the standard therapy might be changed from DOX alone to a combination of DOX plus olaratumab. Presently, however, DOX single agent ought to remain the optimal standard therapy for primary ASTS treatment based on our meta-analysis that included the abovementioned randomized phase II trial.

## Supporting information

S1 Table PRISMA 2009 checklist. (DOC)
